# Organic light-emitting devices based on conducting polymer treated with benzoic acid

**DOI:** 10.1038/s41598-021-82980-0

**Published:** 2021-02-16

**Authors:** Hwa Seung Kang, Dae Hun Kim, Tae Whan Kim

**Affiliations:** 1 Department of Information Display Engineering, Hanyang University, Seoul, 04763 Korea; 2 Department of Electronics and Computer Engineering, Hanyang University, Seoul, 04763 Korea

**Keywords:** Nanoscience and technology, Optics and photonics

## Abstract

We report on the enhanced conductivity of the benzoic-acid-treated poly(3,4-ethlenedioxythiophene):poly(styrene sulfonate) (PEDOT:PSS) electrode for use in highly flexible, organic light-emitting devices (OLEDs). The conductivity of the benzoic-acid-treated PEDOT:PSS electrode increased from 1 to 1583.2 S/cm, in comparison with that of the pristine PEDOT:PSS electrode, due to a complex factor of the H^+^ mole % and the dielectric constant of the benzoic solution. Among the post-treatment methods of the PEDOT:PSS electrodes, the operating voltage at 1000 cd/m^2^ of OLEDs fabricated utilizing the PEDOT:PSS electrode with the benzoic acid treatment has the lowest value, and its maximum luminance is 24,400 cd/m^2^, which are 1.54 and 2.15 times higher than those of OLEDs using PEDOT:PSS electrodes treated with dimethyl sulfoxide and methanol, respectively. The luminance of a flexible OLED with a benzoic-acid-treated PEDOT:PSS electrode after 1400 bending cycles decreased to 83% of the initial luminance, resulting in excellent mechanical stability.

## Introduction

In recent years, the flexible organic light-emitting devices (OLEDs) have received attention as excellent candidates in their potential applications in next-generation wearable devices and multifunctional intelligent systems. In particular, researches on the flexible transparent electrodes have been conducted due to the performance enhancement of the flexible OLEDs. Most transparent electrode for OLEDs have used the indium tin oxide (ITO) due to its excellent properties such as high transparency and high conductivity^1,2^. However, ITO electrode is not suitable as a flexible transparent electrode due to its inherent brittleness^3^. Alternative transparent electrodes such as metallic wires^4–8^, carbon materials^1,9–13^, and conducting polymers^14–18^ have been studied to replace the ITO electrode. Poly (3,4-ethylenedioxythiophene):poly (styrene sulfonate) (PEDOT:PSS) has been widely utilized as a transparent electrode material owing to their outstanding properties of high optical transparency in the visible range, high mechanical stability, and high electrical conductivity^19–22^. However, since the pristine PEDOT:PSS electrode has a very low conductivity compared to other alternative flexible transparent electrodes^23,24^, the performance of OLEDs with PEDOT:PSS electrode is significantly reduced. Therefore, studies to increase conductivity of PEDOT:PSS electrode for high efficient flexible OLEDs is required.

Recently, many studies have been conducted to increase the conductively of PEDOT:PSS electrode. The post treatment of PEDOT:PSS electrode including organic solvent^25,26^, zwitterions^27^, salt^28^ or acid^29–31^ have been reported as alternative to enhance conductivity. The enhancement of conductivity of PEDOT:PSS electrode with the post treatment originate from the reduction of columbic interaction due to phase separation between PEDOT and PSS. Among the various materials, the conductivity of the sulfuric acid treated PEDOT:PSS electrode was reported to be increased more than 3200 S/cm^32^. However, the sulfuric acid, a strong acid that can damage the flexible devices, is not suitable for use as materials to enhance conductivity of PEDOT:PSS electrode^33^. To reduce damage to the flexible device, conducting weak acid treatment on the PEDOT:PSS electrode results in lower conductivity than the strong acid treatment. Therefore, it is necessary to consider mechanisms and process methods that can further improve conductivity of PEDOT:PSS electrode though the weak acid treatment.

In this study, we report mechanism to improve conductivity of PEDOT:PSS electrode with the weak acid treatment for the high efficient OLEDs. The conductivity of PEDOT:PSS with the benzoic acid treatment is enhanced more than 1500 S/cm, and its conductivity enhancement is related to the removal of the insulator PSS in the PEDOT:PSS film due to the hydrogen ion in the benzoic acid solution. In the more detail, the conductivity of PEDOT:PSS with the benzoic acid treatment improves as the variation of parameters such as PH values and dielectric constant. The external quantum efficiency of the flexible OLEDs with the weak acid treatment has almost the same external quantum efficiency as the OLEDs using the ITO electrode.

## Methods

**Figure 1:**
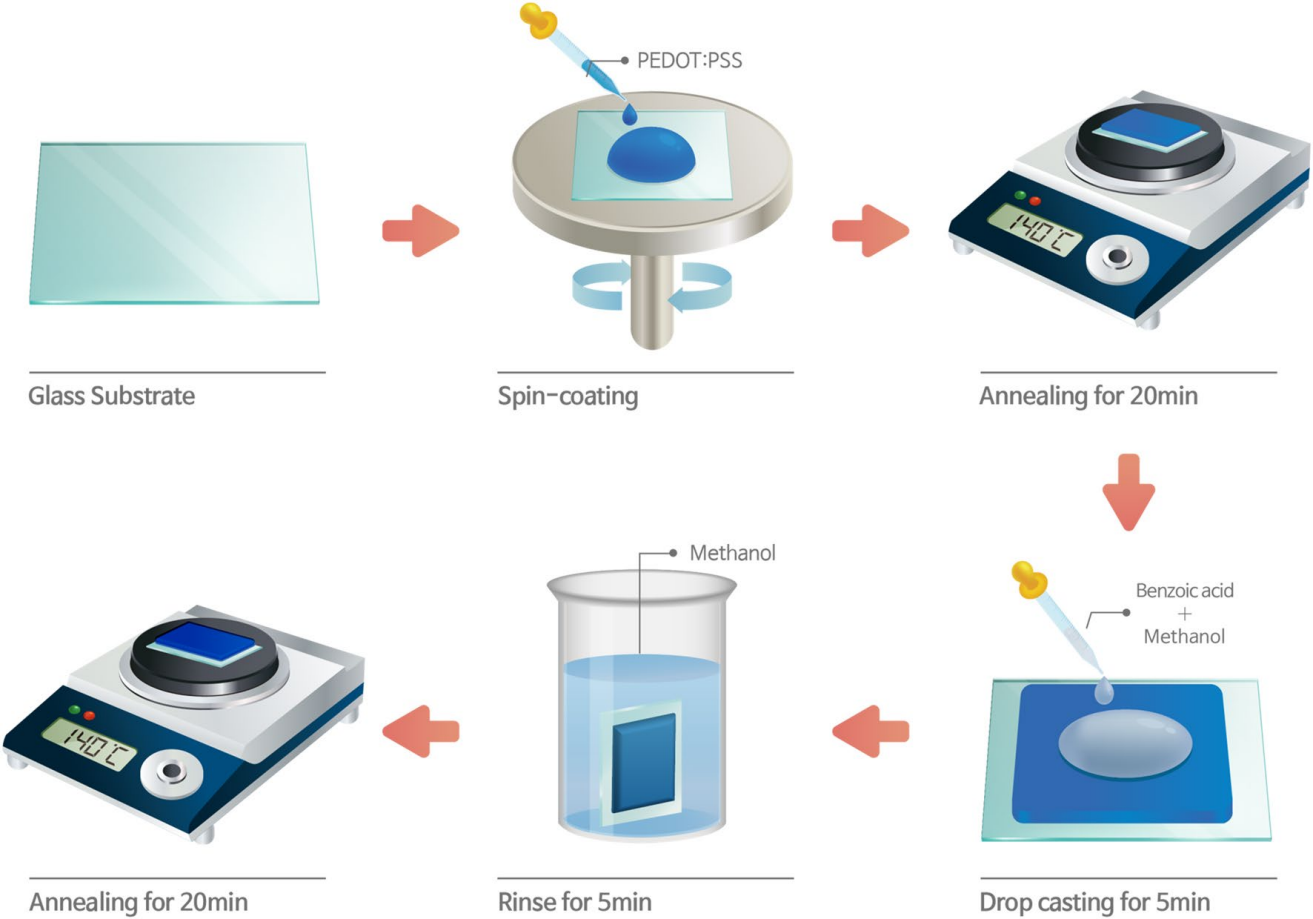
Schematic diagrams of fabrication process for the PEDOT:PSS electrode with benzoic acid treatment (Kang et al.).

The glass substrates were cleaned in acetone, methanol and de-ionized water with ultra-sonication for 10 min, and the cleaned glass substrates were treated by the ultraviolet/ozone lamp for 20 min. A schematic diagram of fabrication process for the PEDOT:PSS with the benzoic treatment was shown in Fig. 1. After the PEDOT:PSS solution was deposed on the cleaned glass substrate by spin-coating at 3000 rpm for 60 s, the PEDOT:PSS film was annealed at 140 °C on a hot plate for 20 min. Then, the benzoic acid powder was mixed a methanol, and then the benzoic acid solution was drop-casting on the PEDOT:PSS film on the hot plate for 5 min at 140 °C. The benzoic acid treated PEDOT:PSS film was rinsed to remove the residual benzoic acid. After the sample had been transferred into an evaporation chamber, the organic layers and the LiF/Al electrodes were deposited at 25 °C and a system pressure of 1.2 × 10^−6^ Torr. The deposition rates of the organic layers and the metal layer were approximately 1.0 and 2.0 Å/s, respectively.

The sheet resistance was measured by using a sheet resistance meter (FPP-40 K, DASOL ENG). The absorption spectra and the transmittance measurements were performed by using an ultraviolet (UV)-visible spectrophotometer (Lambda 650S, Perkin Elmer). The dielectric constant was measured by liquid dielectric constant meter (M871, Nihon Rufuto). The PH is measured by the surface chemical compositions changes were acquired by using X-ray photoelectron spectroscopy (XPS) (K-Alpha + XPS, Thermo Fisher Scientific). The surface roughness, the phase and film thickness of the film were obtained by using atomic force microscopy (AFM) (XE-100, Park Systems). The current density–voltage-luminance characteristics were measured by using a programmable source meter with built-in current and voltage measurement units (M6100, McScience) and a Minolta CS1000 camera. All OLEDs electrical characteristics were measured at the atmosphere conditions.

## Results and discussion

**Figure 2:**
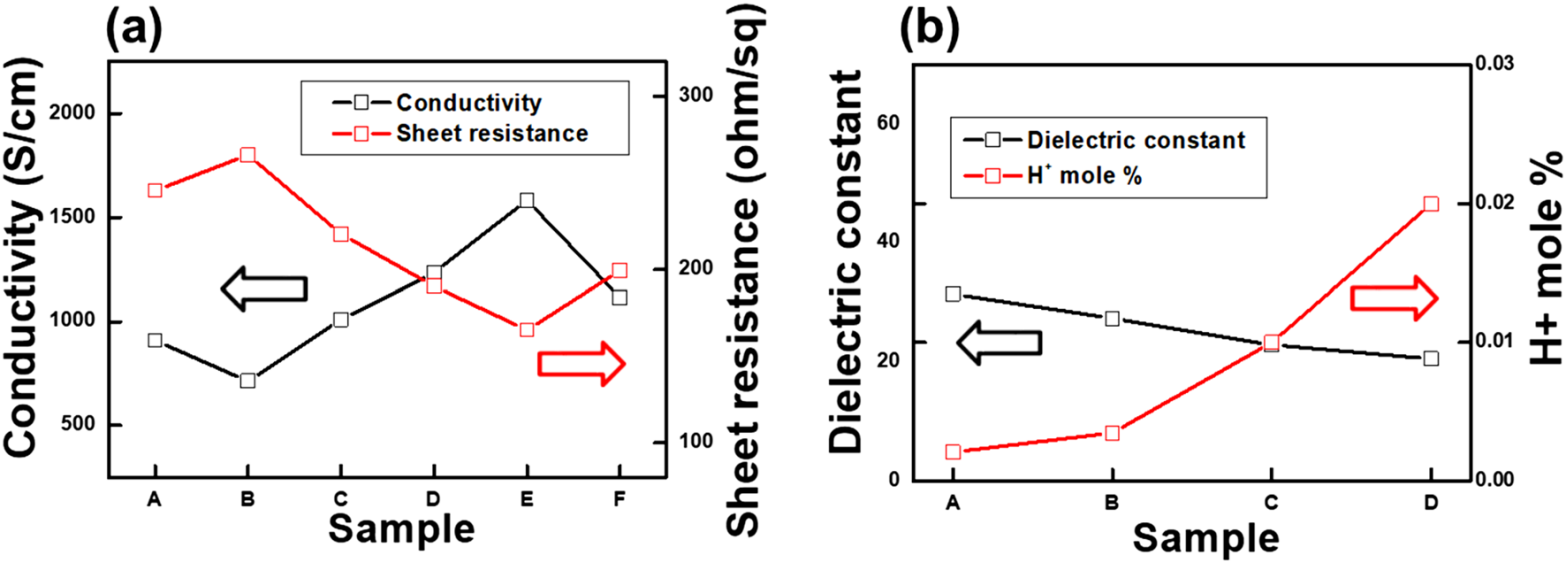
(**a**) Sheet resistances and conductivities of the PEDOT:PSS films with DMSO, methanol and benzoic acid treatments, and (**b**) the dielectric constants and the H + mole % of benzoic acid solution with various weight concentration of the benzoic acid (Kang et al.).

Figure 2a shows the sheet resistances and the conductivities of the PEDOT:PSS films treated with (A) dimethyl sulfoxide (DMSO) or methanol containing (B) 0, (C) 10, (D) 30, (E) 50, and (F) 70 wt.% benzoic acid. The sheet resistances of the PEDOT:PSS films treated with solutions (A) and (B) are 245.5 or 266.1 Ω/sq, and the corresponding conductivities are 909.1 or 714.2 S/cm, respectively. Thus, the corresponding conductivities of the PEDOT:PSS treated with solutions (A) and (B) are improved in comparison with that of the pristine PEDOT:PSS with a conductivity below 1 S/cm^34^. The solutions of the methanol and DMSO have the high dielectric constant. The high dielectric constant of solutions weakens the Columbic interaction between PEDOT and PSS, resulting in an increase of the conductivity of PEDOT:PSS due to the formation of the large PEDOT domain in PEDOT:PSS^23,25^. The sheet resistances of the PEDOT:PSS treated with solutions (C), (D), (E), and (F) are 220.3, 190.4, 162.4 and 199.4 Ω/sq, and the corresponding conductivities are 1010.1, 1234.6, 1583.2 and 1114.8 S/cm, respectively. The conductivities of PEDOT:PSS with benzoic acid treatment are enhanced in comparison with the those of the different organic solvent treatments. The conductivity of PEDOT:PSS with benzoic acid concentration of 50% has highest value in that of the various benzoic acid concentration. For a detailed analysis of the conductivity improvement of the PEDOT:PSS electrode with benzoic acid treatment, the dielectric constants and the H^+^ mole % of benzoic acid solution are measured, as shown in Fig. 2b. The H^+^ mole % of benzoic acid solutions with benzoic acid concentrations of (A) 10, (B) 30, (C) 50, and (D) 70 wt.% are 2.1 × 10^–3^, 3.5 × 10^–3^, 1.0 × 10^–2^, and 2.0 × 10^–2^, respectively, and the dielectric constants of the those solutions with benzoic acid concentrations of (A), (B), (C), and (D) are 31.5, 27.4, 22.9, and 20.6, respectively. The H^+^ mole % of benzoic acid solution gradually increases as the benzoic acid concentration increases. The increases of H^+^ mole % is related to the ionization of benzoic acid solution. Alcohols are amphiprotic substance, much like water, which can act as both an acid and a base by donating or accepting a proton. According to the Brønsted-Lowry definition, methanol can act as a Brønsted base, which can accept a proton^35^. Therefore, the proton can transfer from benzoic acid to methanol, resulting in the formation of the hydrogen ions in the benzoic acid solution. The hydrogen ions in the benzoic acid solution interact the negatively charged PSS, resulting in the formation of the neutral charge PSSH. The Columbic interaction between conductive PEDOT and insulator PSS is reduced, and the electrical conductivity of PEDOT:PSS is enhanced by phase separation between conductive PEDOT and insulator PSS. The dielectric constant is another factor for the enhancement of conductivity, which reduces the Columbic interaction between conductive PEDOT and insulator PSS^36,37^. As a result, the conductivity of the PEDOT:PSS with the benzoic acid treatment increases due to a complex factor of the H^+^ mole % and the dielectric constant of the benzoic solution. Even though the H^+^ mole % and the dielectric constant of benzoic acid at the concentration of 70 wt.% is the highest, it can be seen that the conductivity of the PEDOT:PSS with benzoic acid treatment is lowered due to the residual benzoic acid. The low conductivity benzoic acid residues reduce the conductivity of the PEDOT:PSS.

**Figure 3:**
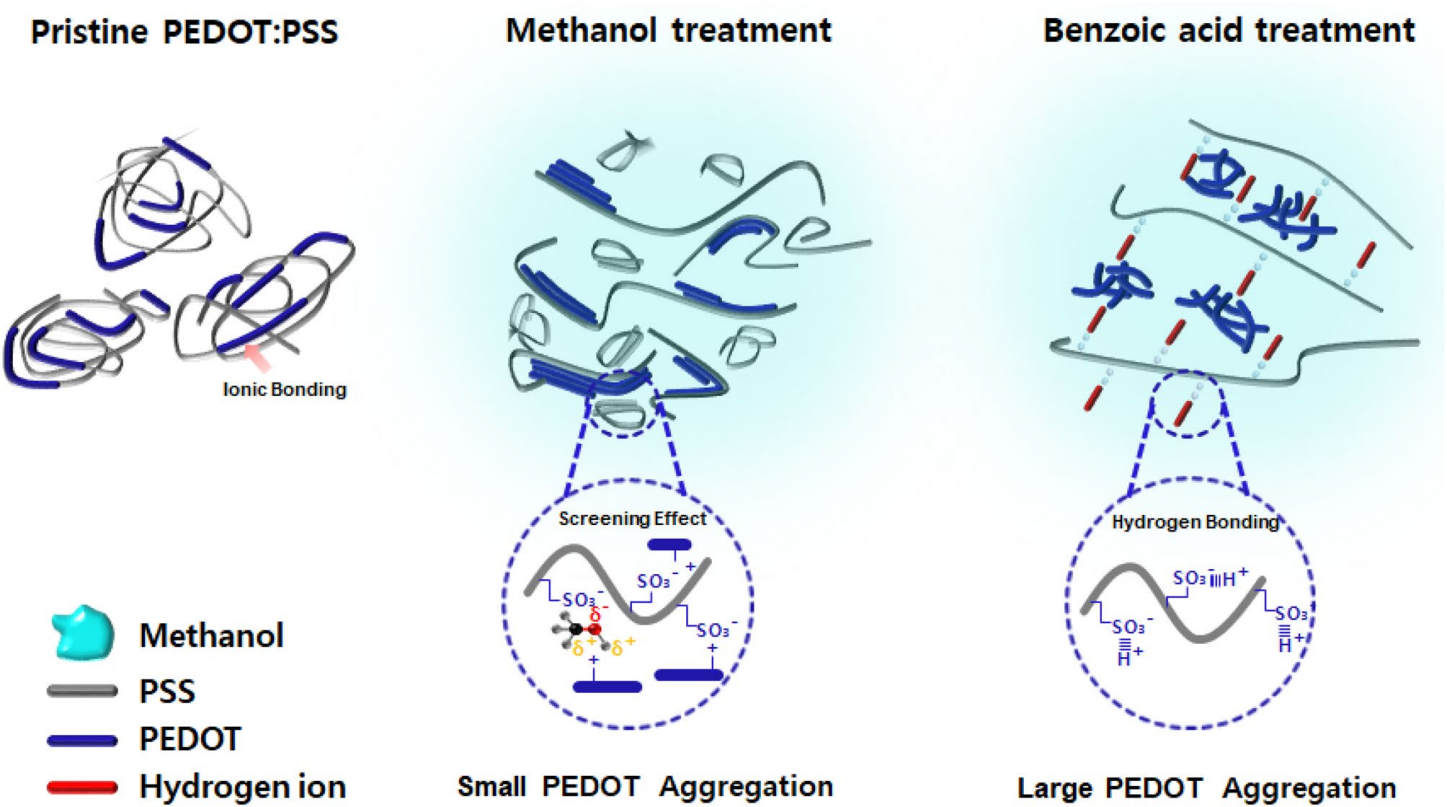
Schematic illustrations of the conductivity enhancement mechanism of PEDOT:PSS film with benzoic acid treatment (Kang et al.).

Figure 3 shows the schematic illustrations of the conductivity enhancement mechanism of PEDOT:PSS in detail. The pristine PEDOT:PSS has core–shell structure composed of ionic bond between the conductive PEDOT and the insulating PSS, and generally contains gel like particles with an insulating PSS shell to stabilize the conductive PEDOT rich particles. The hydrophilic PSS chain is at the outer shell and the hydrophobic PEDOT segments are in the core, which is formed by columbic repulsion among the PSS chains^38,39^. The hydrophilic PSS shell can be used to increase the water solubility of PEDOT:PSS, but the conductivity of PEDOT is reduced because of the impact of the insulting PSS shell. However, after methanol is added to PEDOT:PSS, the methanol, which has a high dielectric constant as a polar solvent, causes a screen effect between the positively charged PEDOT and the negatively charged PSS. When the methanol penetrated into the PEDOT:PSS film, the PSS shell is separated from the PEDOT core due to decrease of the coulomb attraction between the positively charged PEDOT^+^ and the negatively charged PSS^−^, resulting in the formation of the PEDOT aggregation. The increases of conductive PEDOT aggregation means the enhancement of the conductivity of PEDOT:PSS. In the benzoic acid treatment, after the negatively charged PSS^−^ is converted the neutral PSSH due to the hydrogen bonding between PSS^−^ of PEDOT:PSS and the H^+^ ion of benzoic acid solution, the PSSH chains are rinsed away from the PEDOT core, resulting in the reconstruction of shape of an extended PEDOT coil. When the hydrogen bonding effect between H^+^ of benzoic acid and PSS^−^ of PEDOT:PSS is added to the screen effect of the methanol solvent, the region of PEDOT aggregation is increased than that of the methanol solvent treated PEDOT aggregation, which leads to an improvement in conductivity of PEDOT:PSS.

**Figure 4:**
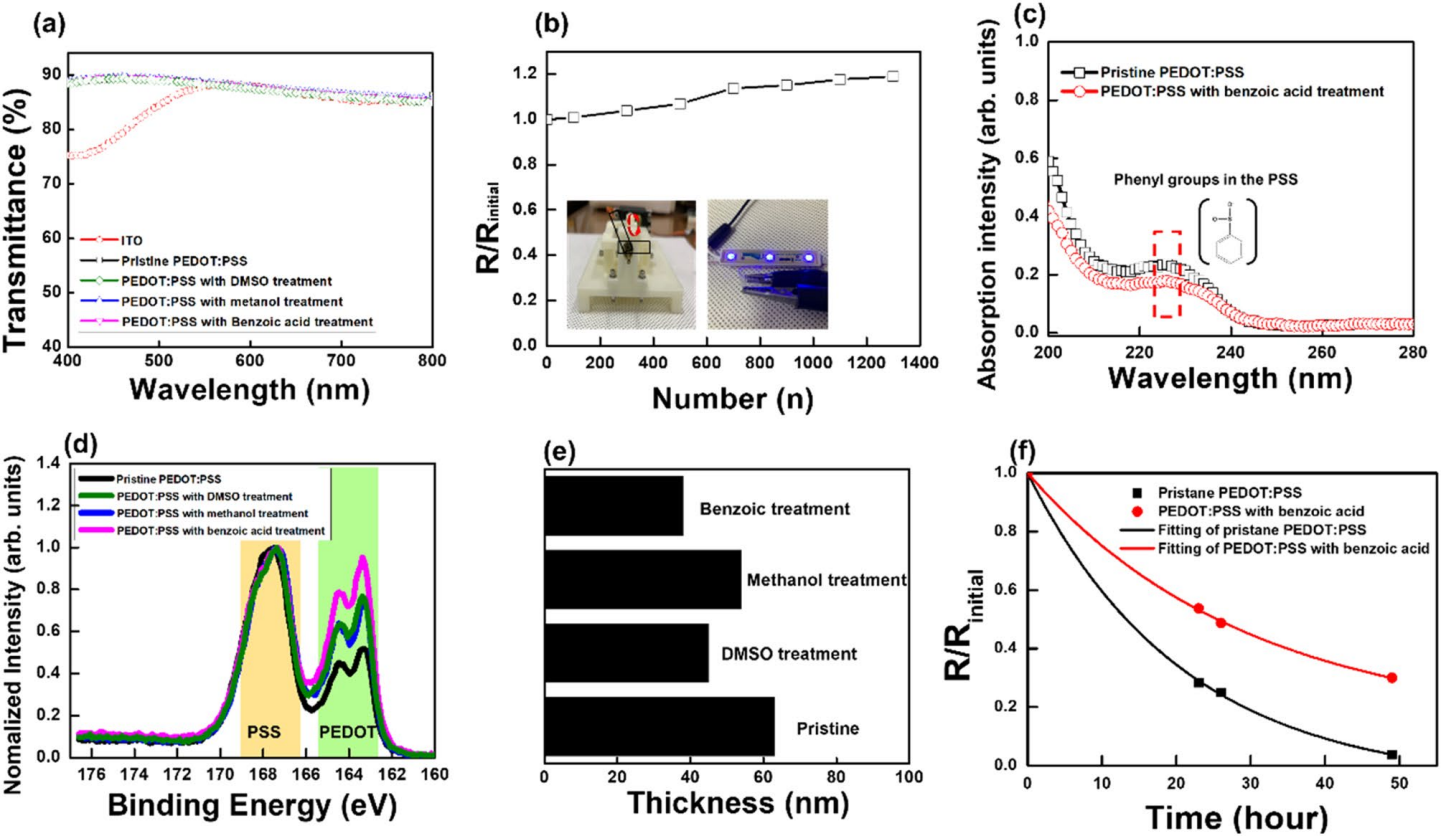
(**a**) Transmittance spectra of the ITO, the pristine PEDOT:PSS, and the PEDOT:PSS with DMSO, methanol, and benzoic acid treatments. (**b**) Bending stability for the PEDOT:PSS electrode with benzoic acid treatment on a PEN substrate. (**c**) Ultra-visible absorption spectra of the PEDOT:PSS films before and after benzoic acid treatment. (**d**) S2p XPS spectra of pristine PEDOT:PSS, and PEDOT:PSS with DMSO, methanol, benzoic acid treatments. (**e**) Film thickness of pristine PEDOT:PSS, and PEDOT:PSS with DMSO, methanol, and benzoic acid treatments. (**f**) Variations in the ratios of the sheet resistances to the initial sheet resistances for the pristine PEDOT:PSS and the PEDOT:PSS with benzoic acid treatment for an 80% humidity (Kang et al.).

The transmittance spectra of PEDOT:PSS films and ITO film in the visible region are shown in Fig. 4a. The intensities of transmittance spectra for the pristine PEDOT:PSS, and the PEDOT:PSS with methanol, benzoic acid, and DMSO treatments have almost the same values in the visible range between 400 and 800 nm. Moreover, the intensity of transmittance spectra for the PEDOT:PSS with benzoic acid treatment is higher than that for the ITO between the 400 nm and 550 nm. The transmittance of the PEDOT:PSS with benzoic acid treatment have high values from 89.59% to 85.17%, which can be used transparent electrodes. Bending properties of the PEDOT:PSS electrode with benzoic acid treatment on poly(ethylene 2,6-naptalate) (PEN) substrate are shown in Fig. 4b. The sheet resistance is increased 1.18 times higher than initial resistance after 1300 cycles of bending at a 5 mm of bending radius. This result shows the excellent mechanical stability of the PEDOT:PSS electrode with benzoic acid treatment after bending process. Figure 4c shows absorption spectra of the PEDOT:PSS films with and without benzoic acid treatment. The absorption peak of PEDOT:PSS film at 225 nm originates from substituted phenyl groups in PSS^40^. The reduction of absorption intensity of PEDOT:PSS after the benzoic acid treatment indicates that the decreases in the amount of PSS from PEDOT:PSS film. Additional confirmation of PSS removal from PEDOT:PSS film after benzoic acid treatment is analyzed using XPS spectrum. Figure 4d shows XPS spectra of pristine PEDOT:PSS or PEDOT:PSS with DMSO, methanol, and benzoic acid treatments. While the S2p peak (S1) at 164 eV originates from the thiophene rings of sulfur atoms in PEDOT, the S2p peak (S2) at 168 eV originates from the sulfonate moiety of sulfur atoms in PSS^41,42^. While the intensity ratio of the S1 to the S2 (S1/S2) of pristine PEDOT:PSS is 2.1, those of the PEDOT:PSS with DMSO, methanol, and benzoic acid treatment are 1.40, 1.35, and 1.09, respectively. The S1/S2 ratio of PEDOT:PSS with benzoic acid treatment is remarkably reduced by 48% than pristine PEDOT:PSS, indicating of peeling of the broken parts of the PSSH chains on the PEDOT:PSS surface. Figure 4e shows that thickness of the pristine PEDOT:PSS, and PEDOT:PSS with DMSO, methanol, and benzoic acid treatments. The thickness of the pristine PEDOT:PSS, and PEDOT:PSS with DMSO, methanol, and benzoic acid treatments are 63, 45, 54, and 38 nm, respectively. The thickness of the PEDOT:PSS films with all treatments are thinner than that of the pristine PEDOT:PSS film. In particular, the thickness of the PEDOT:PSS film after benzoic acid treatment is most reduced from 63 to 38 nm, indicating that the insulating PSSH on the PEDOT:PSS surface can be best removed among various treatment methods. Figure 4f shows the variations in the ratios of the sheet resistance (R_0_) to the initial sheet resistance (R_i_) for the pristine PEDOT:PSS electrodes and for the PEDOT:PSS with benzoic acid treatment (70%) at 80% humidity. When the ratios of R_0_/ R_i_ for the pristine PEDOT:PSS electrodes and for the PEDOT:PSS electrodes with benzoic acid treatment are 1/2, their lifetimes are 13.3 and 25.7 h, respectively. Because the PEDOT:PSS electrode absorbs moisture from the atmosphere, the sheet resistances of the pristine PEDOT:PSS films sharply increase over time. However, the sheet resistance of the PEDOT:PSS electrode with benzoic acid treatment slowly increases in comparison with that of the pristine PEDOT:PSS electrode. The stability under humidity of the PEDOT:PSS electrode with benzoic acid treatment is enhanced because the amount of PSS at the surface that absorbs moisture is reduced by the benzoic acid treatment and because the more compact structural rearrangement of the PEDOT:PSS with benzoic acid treatment improves stability under humid conditions^43,44^. The improved stability of the PEDOT:PSS electrode with benzoic acid treatment can increase the lifetime of the OLEDs based on such electrodes.

**Figure 5:**
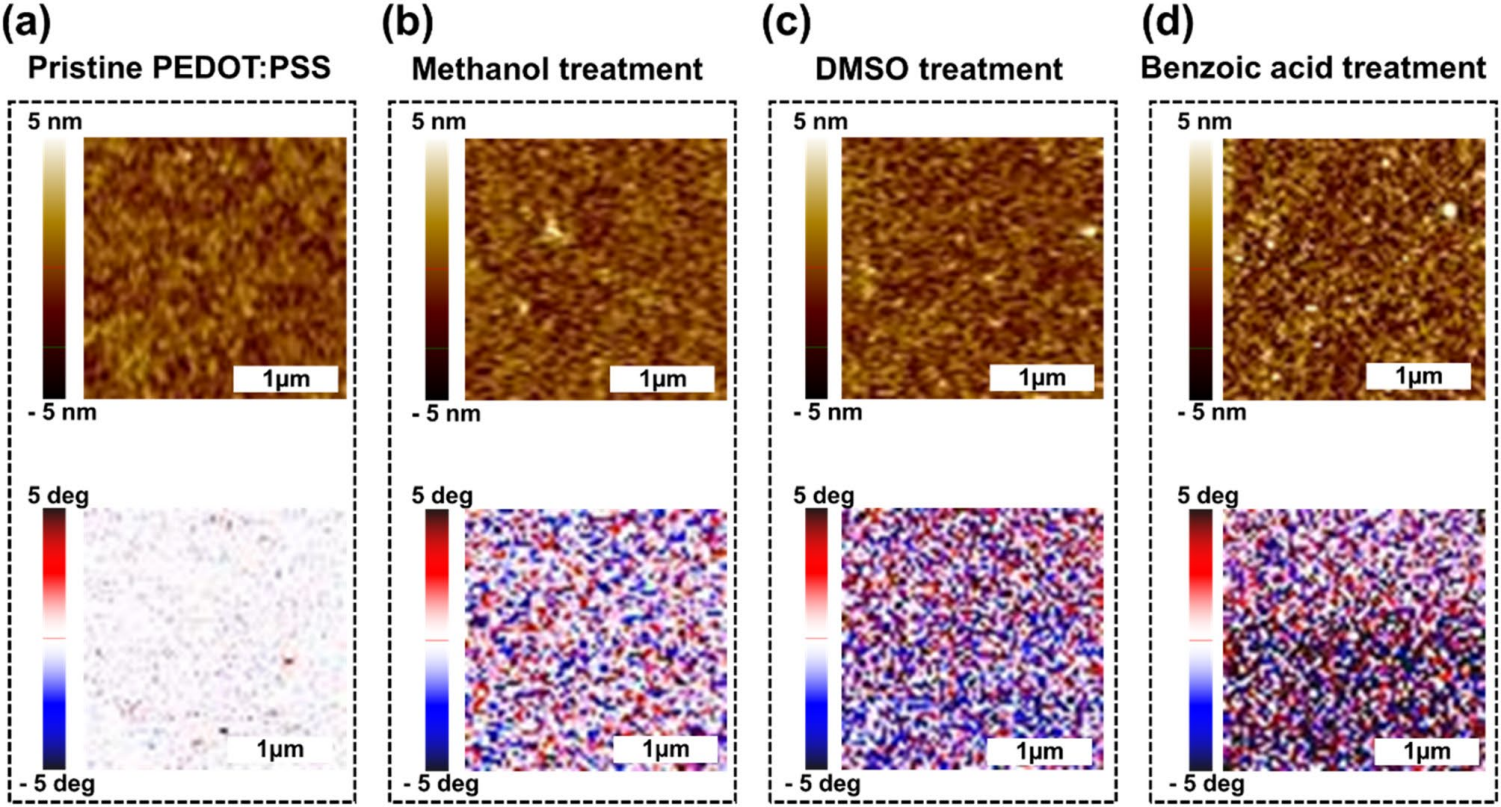
Atomic force microscope topography images of (**a**) pristine PEDOT:PSS, and PEDOT:PSS with (**b**) methanol, (**c**) DMSO, and (**d**) benzoic acid treatments, and phase images of (**e**) pristine PEDOT:PSS, and PEDOT:PSS with (**f**) methanol, (**g**) DMSO, and (**h**) benzoic acid treatments (Kang et al.).

Figure 5 shows the AFM topography and phase images of (a, e) pristine PEDOT:PSS, and PEDOT:PSS with (b, f) methanol, (c, g) DMSO, and (d, h) benzoic acid treatments. When the root-mean-square (RMS) roughness of pristine PEDOT:PSS film is 0.88 nm, the RMS roughness of PEDOT:PSS films with methanol, DMSO, and benzoic acid treatment are 0.97, 1.08, and 1.59 nm, respectively. The RMS value of PEDOT:PSS benzoic acid treatment has the largest RMS value of all PEDOT:PSS samples. The insulating PSS shells become separated from the conductive PEDOT chain with increasing screening effect and H^+^ molar %, thereby increasing the aggregation region of the conductive PEDOT. An increase in the aggregation region of the conductive PEDOT is indicated by an increases in the RMS value, which means an increase in conductivity of PEDOT:PSS film with benzoic acid treatment.

For more reliable analysis, the AFM phase measurement was performed on the PEDOT:PSS films. The associated variation in contrast in the phase images is related to the change in the chemical composition of the PEDOT:PSS film. While the red region can be assigned to a relatively hard phases attributed to the PEDOT rich region in measured phase images under tapping mode, the blue region related to the lower phases indicates the excess of PSS^45–47^. The phase image of the pristine PEDOT:PSS film does not show a distinct change in contrast, as shown in Fig. 5e. This means that the PEDOT chain mixes well with the PSS chain, and the film is mostly covered with PSS rich domain. When the PSSH chains are separated on the surface of the PEDOT:PSS film due to the various post treatments, the shape of the extended PEDOT coil on the surface is reconstructed resulting in the increase of the phase variation^48,49^. The phase variation of the PEDOT:PSS with benzoic acid treatment is more distinct than those of the PEDOT:PSS with methanol and DMSO treatments, as shown in Fig. 5f–h. While the increase of PEDOT rich regions of PEDOT:PSS with methanol and DMSO treatment originates from the screening effect, the increase in PEDOT:PSS with benzoic acid treatment is caused not only by the screening effect, but also by an increase in the amount of H^+^ ions. Therefore, in the benzoic acid treatment, the loss of the PSSH on the PEDOT:PSS increases compared to other post treatment methods resulting in the increase of the conductive PEDOT rich regions.

**Figure 6:**
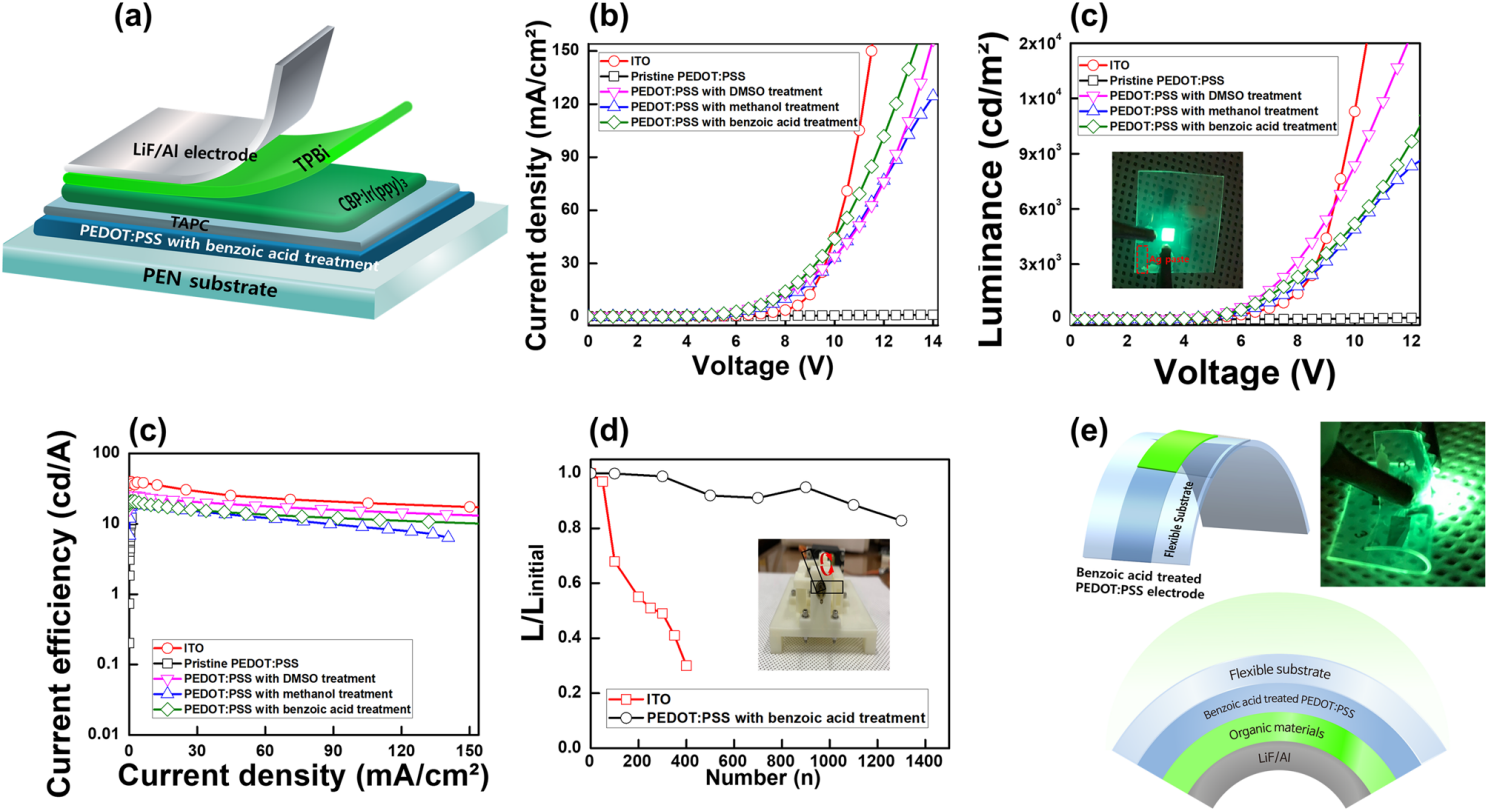
(**a**) Schematic diagram, (**b**) current density – voltage, (**c**) luminance – voltage, (**d**) current efficiency – current density, (**e**) bending stability of flexible OLEDs, (**f**) schematic illustrations of bending test and photographic images of flexible OLED using the PEDOT:PSS electrode with benzoic acid treatment (Kang et al.).

Figure 6a shows a schematic diagram of the structure of the OLEDs utilizing the PEDOT:PSS with benzoic acid treatment. The device structure is glass substrate/PEDOT:PSS anode with benzoic acid treatment (38 nm)/1,1-Bis[(di-4-tolylamino)phenyl]cyclohexane (TAPC) hole-transport layer (HTL) (40 nm) /4,4′-Bis(N-carbazolyl)-1,1′-biphenyl (CBP):Ir(ppy)_3_ emitting layer (EML) (30 nm)/2,2′,2″-(1,3,5-Benzinetriyl)-tris(1-phenyl-1-H-benzimidazole) (TPBi) electron transport layer (ETL) (30 nm)/ LiF electron injection layer (EIL) (1 nm)/Al cathode (100 nm). Figure 6b shows the current density—voltage characteristics for OLEDs using ITO, and PEDOT:PSS with DMSO, methanol, and benzoic acid treatments. The OELDs with pristine PEDOT:PSS electrode has a current density of less than 1 mA/cm^2^ due to the low conductivity of the pristine PEDOT:PSS electrode. The operating voltages at a current density of 10 mA/cm^2^ of the OLEDs using the PEDOT:PSS electrode with DMSO, methanol, and benzoic acid treatments are 7.8, 8.0, and 7.5 V, respectively. The luminance—voltage characteristics for OLEDs using ITO, and PEDOT:PSS with DMSO, methanol, and benzoic acid treatments are shown in Fig. 6c. The pristine PEDOT:PSS electrode could not be operated at luminance up to a luminance of 100 cd/m^2^ due to the low conductivity of the PEDOT:PSS electrode. The operating voltages at a luminance of 1000 cd/m^2^ of the OLEDs using the PEDOT:PSS electrode with DMSO, methanol, and benzoic acid treatments are 6.7, 7.1, and 5.9 V, respectively. The high conductivity of the PEDOT:PSS electrode with benzoic acid treatment can facilitate the current flow in the OLED, which leads to an enhancement in the current density. Among the post-treatment methods of PEDOT:PSS electrodes, the operating voltage at 1000 cd/m^2^ of OLEDs using the PEDOT:PSS electrode with the benzoic acid treatment is the lowest, and its maximum luminance is 24,400 cd/m^2^, which indicates 1.54 and 2.15 times higher than those of OLEDs using the PEDOT:PSS electrode with the DMSO and methanol treatment, respectively, because of the higher conductivity of PEDOT:PSS electrode with benzoic acid treatment than other post treatments. Figure 6d shows the current efficiency—current density characteristics for OLEDs. The current efficiency is related to the charge balance of hole and electron in EML of OLEDs. The maximum current efficiencies of the OLED using PEDOT:PSS electrode with DMSO, methanol, and benzoic acid treatments are 20.9, 18.8, and 25.3 cd/A, respectively. The current efficiency of OLEDs using PEDOT:PSS electrode with treated benzoic acid treatments is higher than those of OLEDs using PEDOT:PSS electrode with DMSO and methanol treatments due to the higher conductivity of PEDOT:PSS electrode with treated benzoic acid treatment. The maximum current efficiencies of the OLED using the PEDOT:PSS electrode with benzoic acid treatment and of the OLED with an ITO electrode are 25.3 and 39.8 cd/A, respectively. The maximum current efficiency of the OLED using the PEDOT:PSS electrode with benzoic acid treatment is smaller than that of the OLED with an ITO electrode due to the high conductivity and the smooth surface morphology of the ITO electrode. However, the PEDOT:PSS electrode is still better than the ITO electrode for use in flexible and transparent OLEDs due to its strength, flexibility, and durability. Figure 6e shows the bending stability of the flexible OLEDs using the ITO electrode and the PEDOT:PSS electrode with benzoic acid treatment at 5 mm of the bending radius. The luminance of the flexible OLEDs based on ITO electrode is 30% of the initial luminance value after 400 bending cycles. The surface of the ITO electrode can form the crack after bending due to inherent brittleness of ITO, resulting in a sharply decrease in the luminance of the flexible OLED. On the other hand, the flexible OLED using PEDOT:PSS electrode with benzoic acid treatment is 83% of the initial luminance after 1400 bending cycles. The schematic illustrations of the bending test for flexible OLEDs, which use the inner bending method, and the photograph of the flexible OLED using PEDOT electrode with benzoic acid treatment after bending are shown in Fig. 6f. When the flexible OLEDs using PEDOT:PSS with benzoic acid treatment is bent to a radius of 5 mm, the flexible OLEDs using PEDOT:PSS with benzoic acid treatment operates well. In the inner bending process, flexible OLEDs might have a small decrease in luminance due to the locally delaminated PEDOT:PSS on the PEN substrate^50^, the increase of sheet resistance is very small, resulting in the excellent bending stability of the PEDOT:PSS electrode with benzoic acid treatment.

## Conclusion

We report on the conductivity enhancement in the PEDOT:PSS electrode with benzoic acid treatment for application in highly efficient, flexible OLEDs. The conductivity of PEDOT:PSS with benzoic acid treatment enhances up to 1538 S/cm due to the increases of the high dielectric constant and an amount of the H^+^ ion of the benzoic acid solution. The insulating PSS shells become separated from the conductive PEDOT core with increasing the dielectric constant and H^+^ molar %, thereby increasing the aggregation region of the conductive PEDOT. While the operating voltages of the OLED using the PEDOT:PSS electrode with benzoic acid treatment at a luminance of 1000 cd/m^2^ is 5.9 V, the maximum current efficiency is 25.3 cd/A, which is much better than OLEDs using PEDOT:PSS electrode with DMSO and methanol treatment. The enhanced performances of OLEDs using the PEDOT:PSS electrode with benzoic acid treatment is attributed to an increase the conductivity of PEDOT:PSS film. The flexible OLED using the PEDOT:PSS electrode with benzoic acid treatment is 83% of the initial luminance after 1400 bending cycles at a bending radius 5 mm. The PEDOT:PSS electrode with benzoic acid treatment can be used as an alternative high conductive, transparent electrode for flexible OLEDs.
